# A Rare Case of Metachronous Tumor: Recurrent Primary Lung Cancer

**DOI:** 10.7759/cureus.73536

**Published:** 2024-11-12

**Authors:** Hassan Edward Bakali, Seda Kahraman, Ayse Gul Ergonul, Dilara Ozyigit Buyuktalanci

**Affiliations:** 1 Thoracic Surgery, Ege University Faculty of Medicine, Izmir, TUR; 2 Thoracic Surgery, Democracy University, Buca Seyfi Demirsoy Teaching and Research Hospital, Izmir, TUR; 3 Pathology: Department of Medical Pathology, Başakşehir Çam ve Sakura City Hospital, Istanbul, TUR

**Keywords:** copd (chronic obstructive pulmonary disease), metachronous lung tumors, multiple primary lung cancer, synchronous lung tumors, vats, wedge resection

## Abstract

Multiple primary lung cancers (MPLC) are defined as lung cancers that develop at the time of primary lung cancer detection (synchronous) or during the follow-up period (metachronous). The incidence of MPLC is increasing due to the early detection of lesions by widespread screening methods, improved surgical, medical, and radiation treatment options, and increased survival. We present a case of a patient who was operated on in our clinic and diagnosed with primary lung cancer for the third time with different histopathology. Although rare, we wanted to emphasize that the follow-up and treatment planning for MPLCs, whose incidence is increasing, should be a multidisciplinary approach.

## Introduction

Lung cancers found at the time of primary lung cancer (PLC) detection or during follow-up after curative treatment are referred to as multiple primary lung cancers (MPLC). The North American Association of Central Cancer Registries (NAACCR) classifies multiple primary malignancies as either synchronous (i.e., at the same time of primary malignancy detection) or metachronous (i.e., six months after the initial diagnosis) [1,2]. The incidence of MPLC is increasing due to the early detection of lesions by advanced and widespread screening methods [2]. Improved quality of life due to minimally invasive surgery and the vast improvements in medical and radiation treatment options have also improved the overall survival of lung cancer patients, leading to an increase in the incidence of MPLC [3]. We present the case of a patient who had two previous surgeries for primary lung carcinoma of different histopathology and was operated on for the third time in our clinic with a new diagnosis of primary lung cancer.

This article was presented as a poster at the 12th National Congress of the Turkish Society for Thoracic Surgery (19-22 October 2023, La Blanche Island, Bodrum, Türkiye).

## Case presentation

**Figure 1:**
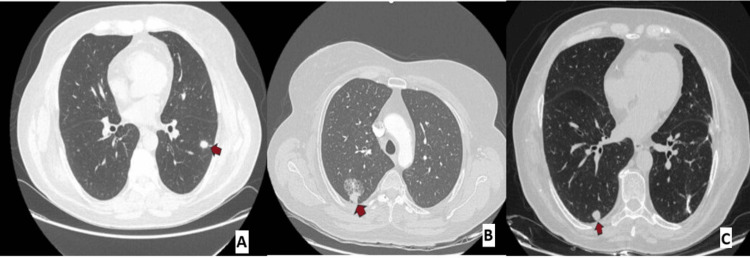
Thoracic computed tomography (CT) showing the patient's lesions over the years (highlighted by red arrows) A: 13 mm nodule in the lower lobe of the left lung on thorax CT, B: 34 mm ground-glass lesion with a solid component in the posterior upper lobe of the right lung on thorax CT, C: 10 mm solid nodule in the posterior lower lobe of the right lung on thorax CT

**Figure 2:**
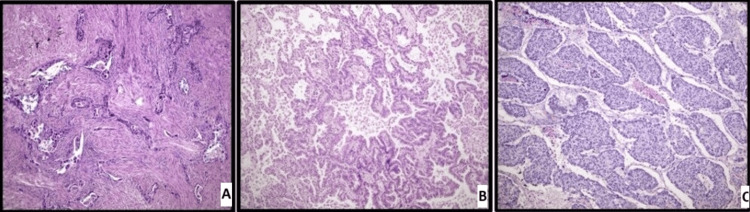
Histopathology images of the patient's tumors stained with hematoxylin and eosin (H&E) A: Squamous cell carcinoma islands spreading as irregular infiltrative islands within the desmoplastic stroma (H&E, x100), B: Adenocarcinoma structures showing lepidic, acinar, and micropapillary pattern (H&E, x100), C: Nests of large cell neuroendocrine carcinoma with neuroendocrine cell morphology in solid and trabecular arrangement (H&E, x100)

A 72-year-old woman with chronic obstructive pulmonary disease (COPD) and a 50-pack-year active smoking history was evaluated with a computerized tomography (CT) scan after she complained of a chronic cough. On her chest CT, a 13-mm nodule in the lower lobe of the left lung was detected, and she subsequently underwent videothoracoscopic (VATS) wedge resection of the lesion (Figure 1). Pathological evaluation revealed it as squamous cell carcinoma, and the patient was staged as T1bN0M0, according to the 8th edition TNM staging for lung cancer (Figure 2). Due to the patient’s clear surgical margins and comorbidities, no further surgical or medical treatment was recommended. During her follow-up two years later in 2018, a 34 mm ground-glass opacity lesion with a solid component was detected in the upper lobe of the right lung on chest CT (Figure 1). A second surgery was thus performed, an upper lobe wedge resection via thoracotomy. Pathology revealed a lepidic dominant adenocarcinoma (Figure 2). The patient was followed up, and again, no adjuvant treatment was planned. In 2023, a 10 mm nodule in the lower lobe of the right lung was seen on the patient’s routine follow-up CT scan (Figure 1), and wedge resection was performed via thoracotomy to resect the lesion. Pathological examination revealed it as a large cell neuroendocrine carcinoma (LCNEC) (Figure 2). With the diagnosis of a third metachronous primary lung cancer, the patient was evaluated by an expert multidisciplinary oncology board, which included pathologists, radiologists, pulmonologists, and medical, surgical, and radiation oncologists from our university hospital. Adjuvant chemotherapy treatment was planned for the patient.

Although smoking cessation was recommended to the patient starting from the time her first lesion was discovered in 2016, she continued smoking and remains an active smoker to this day.

## Discussion

In patients with PLC, the histopathologic type, medical history, and detailed radiologic and nuclear studies are important in evaluating lesions as recurrent, metachronous, or metastatic disease [2,3]. The criteria developed by Martini-Melamed (1975) and modified by Antakli (1995) serve to guide patient evaluation and management [2,3]. Some studies in the literature have also shown molecular discrepancies between PLC and metastases, especially in EGFR, K-RAS, p53, and ALK mutations, which can be used to distinguish the two [4]. Similar cases of synchronous and second metachronous cancer are rarely reported in the literature [3,5,6]. Cases of a third metachronous lung cancer with completely different histopathology are even rarer.

Surgical treatment is still the gold standard treatment for early-stage primary lung cancer [4-7]. Although lobectomy is the standard for early-stage PLC, sublobar resections should be considered in cases where the second lesion is in the same lung; a pneumonectomy, being a morbid surgery, has been shown to be a negative prognostic factor [7,8]. Patients with poor pulmonary reserve also benefit from sublobar resections, especially segmentectomy, which has been shown to have similar long-term oncologic outcomes to lobectomy [8,9].

Adjuvant chemotherapy has also been shown to improve overall survival. Medically inoperable patients or patients with advanced disease can also benefit from chemotherapy, radiotherapy, immunotherapy, or an appropriate combination of these treatments. Ablation has also been shown to be effective in local control of tumors [10]. It is worth noting that without adjuvant chemotherapy or radiotherapy, the histopathologically distinct tumors in our patient developed two and five years apart. Smoking, being a known risk factor for primary lung carcinoma, may have contributed to the patient’s MPLC incidence. Rzechonek et al., in their study of the clinical characteristics of metachronous lung cancer, identified adenocarcinoma histopathology and non-anatomic resection as risk factors for metachronous lung cancer [6]. Soro-García et al. also reported non-anatomical resections as a risk factor for MPLC [3]. Wedge resections have been associated with a high risk of recurrence compared to their anatomical resection counterpart, segmentectomy [11].

In our patient's case, the lack of multimodal treatment during the first two occurrences of her MPLC might have also contributed to the development of a third metachronous lung cancer. The patient also had non-anatomic lung resections, which have been associated with a higher risk for recurrence. Many of the cases in the literature mostly describe two synchronous or metachronous primary lung cancers. Damania et al. described a case of three metachronous primary lung cancers in 2020 [12]. In their case, the patient developed two squamous cell carcinomas and a small cell carcinoma the third time. Similar to our case, their patient was also a chronic active smoker, to which they attributed the MPLC. They highlighted the importance of smoking cessation and long-term surveillance. Our case is distinctively unique in that the patient developed three histopathologically different non-small cell carcinomas.

## Conclusions

Due to improved screening methods like low-dose CT, MPLC cases are on the rise. Their treatment and follow-up require multimodal and multidisciplinary intervention. All treatment modalities, including immunotherapy, should be considered for appropriate patients.
